# The Spleen Virome of Australia’s Endemic Platypus Is Dominated by Highly Diverse Papillomaviruses

**DOI:** 10.3390/v17020176

**Published:** 2025-01-26

**Authors:** Subir Sarker, Saranika Talukder, Ajani Athukorala, Pam L. Whiteley

**Affiliations:** 1 Biomedical Sciences & Molecular Biology, College of Medicine and Dentistry, James Cook University, Townsville, QLD 4811, Australia; 2 Australian Institute of Tropical Health and Medicine, James Cook University, Townsville, QLD 4811, Australia; 3 Department of Microbiology, Anatomy, Physiology and Pharmacology, School of Agriculture, Biomedicine and Environment, La Trobe University, Melbourne, VIC 3086, Australia; ajaniathukorala@gmail.com; 4 College of Science and Engineering, James Cook University, Townsville, QLD 4811, Australia; saranika.talukder@jcu.edu.au; 5 Melbourne Veterinary School, The University of Melbourne, Werribee, VIC 3030, Australia; pamw@unimelb.edu.au

**Keywords:** monotreme, platypus, virome, metagenomics, phylogenetics

## Abstract

The platypus (*Ornithorhynchus anatinus*), a unique monotreme, represents a pivotal point in mammalian evolution with its distinctive traits, such as electroreception and venom production. Despite its evolutionary significance, the viral diversity within platypuses remains poorly understood. This study employed next-generation sequencing to investigate the virome of the dead platypuses, uncovering a range of novel and divergent viruses. Among the identified viruses were four complete genomes of papillomaviruses (OaPV1–4) exhibiting substantial divergence from known strains, suggesting a novel genus within the subfamily *Secondpapillomavirinae*. Additionally, five novel parvoviruses were detected, including two with complete genomes, highlighting the complex viral ecosystem of the platypus. Phylogenetic analysis placed these viruses in unique evolutionary branches, further demonstrating the platypus’s evolutionary significance. A circular DNA virus, a tombus-like virus, and a nodamuvirus were also identified, expanding the understanding of viral diversity in monotremes. These findings offer crucial insights into viral evolution in one of the most unique mammalian lineages, emphasising the need for further exploration to assess ecological and pathological impacts on platypus populations.

## 1. Introduction

The platypus (*Ornithorhynchus anatinus*) is one of the most enigmatic mammals, representing a key evolutionary link within the ancient monotreme lineage that diverged from therian mammals approximately 166 million years ago [1]. This egg-laying species exhibits a suite of unique biological traits, including electroreception and venom production, underscoring its critical role in understanding mammalian evolution [2,3]. Despite its evolutionary and ecological significance, research into the virome of the platypus remains limited, leaving substantial gaps in our knowledge of the viruses that may infect this iconic species.

Metagenomics or metatranscriptomics has been pivotal in advancing our understanding of viral diversity [4,5,6,7,8]. By enabling the analysis of entire microbiomes, including bacterial, viral, and host genomes, metagenomic approaches provide a comprehensive view of viromes from complex biological samples [7,8,9,10,11,12,13,14,15]. The technique utilises either long-read or short-read sequencing platforms, such as the Illumina system, which offers high sensitivity, accuracy, and quantitative capabilities at a reduced cost. Computational tools further facilitate the assembly and characterisation of viral genomes, even in the absence of complete reference data [16]. However, challenges remain, particularly the limited representation of viral sequences in genomic databases and the inherent genomic diversity of viruses, which complicates their detection and evolutionary analysis.

Viruses are ubiquitous across vertebrate hosts, including mammals, reptiles, and birds [9], yet studies investigating viral diversity in the platypus are scarce. Advances in next-generation sequencing (NGS) technologies and viral metagenomics have revolutionised the detection and characterisation of both known and novel viruses across diverse taxa, including underexplored or cryptic hosts [16]. Viral families such as *Parvoviridae*, *Papillomaviridae*, and *Circoviridae* have been identified in mammals and other vertebrates, often demonstrating host adaptation, ecological niche specialisation, and significant pathogenic potential [17,18,19,20,21,22]. These discoveries raise important questions about the presence, diversity, and potential impacts of similar viruses in monotremes, including the platypus.

Understanding viral diversity in the platypus is essential for multiple reasons. First, as a basal mammalian lineage, the platypus may harbor viruses with unique evolutionary origins, offering critical insights into the coevolution of viruses and their hosts. Second, establishing baseline data on viral infections is crucial for assessing potential risks to platypus populations, particularly in the face of increasing habitat loss, climate change, and environmental pressures [23]. Finally, characterising novel viral lineages in the platypus can contribute to our broader understanding of viral evolution, host–pathogen dynamics, and cross-species transmission across vertebrates [9,24].

This study aims to address these knowledge gaps by investigating the viral diversity of the dead platypus using advanced genomic tools. Through the detection and characterisation of novel and divergent viruses, this work seeks to elucidate the evolutionary roles of viruses in one of Earth’s most unique mammals.

## 2. Materials and Methods

### 2.1. Sampling and Ethical Consideration

The samples were obtained during pathological examination of dead platypuses (*Ornithorhynchus anatinus*) received from Wildlife Health Victoria, Australia (Table 1). The animals, found dead at six locations across Victoria, were transported to the Melbourne Veterinary School in Werribee for pathological and toxicological assessments to investigate potential causes of death. However, their health status prior to death and the definitive causes of mortality remained inconclusive. Whenever possible, samples were stored at −80 °C, but the duration for which the carcasses had been exposed to environmental conditions prior to collection was not documented. While animal ethics approval was not required, the La Trobe University Animal Ethics Committee approved the use of these diagnostic materials for publication as part of a surveillance program.

### 2.2. Virus Enrichment and Virus Nucleic Acid Extraction

To remove potential contaminants such as host cells, bacteria, and free nucleic acids from the tissue samples, viral particles were enriched following modified versions of established protocols [8,25]. In summary, tissue samples were sectioned with a sterile scalpel blade and placed into Eppendorf tubes containing phosphate-buffered saline (PBS). A bead was added to each tube, and the samples were subjected to vigorous vortexing for approximately 10 min using a TissueLyser-LT2 (Qiagen, Hilden, Germany) to achieve complete tissue homogenisation. The homogenised samples were centrifuged at 17,000× *g* for three minutes, and the resulting supernatant was filtered through a 0.80 µm syringe filter. The filtrate underwent additional processing, including ultracentrifugation at 178,000× *g* for one hour at 30 PSI and 4 °C using a Hitachi Ultracentrifuge CP100NX. The supernatant was discarded, and the pellet was resuspended in 130 µL of sterile PBS. To eliminate residual nucleic acids, 2 µL of benzonase nuclease (25–29 U/µL, >90% purity, Merck KGaA, Darmstadt, Germany) and 1 µL of micrococcal nuclease (2,000,000 gel U/mL, New England Biolabs, Ipswich, MA, USA) were added to the filtrate, followed by incubation at 37 °C for two hours. The enzymatic reaction was terminated by adding 3 µL of 500 mM ethylenediaminetetraacetic acid (EDTA). Viral nucleic acids were extracted using the QIAamp Viral RNA Mini Kit (Qiagen, Valencia, CA, USA) without carrier RNA, enabling the simultaneous extraction of both viral DNA and RNA. The extracted nucleic acids were then evaluated for concentration and integrity using a Nanodrop spectrophotometer and an 4150 Agilent TapeStation (Agilent Technologies, Mulgrave, VIC, Australia) at the Genomic Platform of La Trobe University.

### 2.3. Next-Generation Sequencing

Before constructing the libraries, cDNA synthesis was performed on the extracted RNA, followed by amplification using the Whole Transcriptome Amplification Kit (WTA2, Sigma-Aldrich, Darmstadt, Germany), as per the manufacturer’s instructions. The resulting PCR products were purified using the Wizard^®^ SV Gel and PCR Clean-Up Kit (Promega, Madison, WI, USA). The concentration and quality of the purified products were assessed using the Qubit dsDNA High Sensitivity Assay Kit and a Qubit Fluorometer v4.0 (Thermo Fisher Scientific, Waltham, MA, USA).

Library preparation for individual samples was conducted with the Illumina DNA Prep Kit (Illumina, San Diego, CA, USA) following the manufacturer’s protocol. An initial input of 250 ng of DNA, quantified with the Qubit Fluorometer v4.0, was used for library construction. The Australian Genome Research Facility (AGRF) in Melbourne, Australia, evaluated the quality and concentration of the prepared libraries. The libraries were normalised and pooled in equimolar amounts. The quality and concentration of the final pooled library were reevaluated before sequencing using the same methods. Cluster generation and sequencing were carried out at AGRF on the Illumina^®^ NovaSeq platform, producing 150-bp paired-end reads in accordance with the manufacturer’s guidelines.

### 2.4. Bioinformatic Analyses

Sequencing data were analysed using an established workflow [5,25,26,27] implemented in Geneious Prime (version 2023.1.1, Biomatters, Auckland, New Zealand). An initial quality assessment of the raw reads was performed, followed by preprocessing steps to eliminate ambiguous base calls, low-quality reads, and Illumina adapter sequences. The trimmed reads were then aligned to the platypus genome (*Ornithorhynchus anatinus*, accession no. GCA_004115215.2) to remove potential host DNA contamination. Subsequently, the reads were mapped to the *Escherichia coli* genome (GenBank accession no. U00096) to exclude bacterial contamination. The filtered, unmapped reads were subjected to de novo assembly using the SPAdes assembler (version 3.10.1) [28], with the ’careful’ setting on the LIMS-HPC system, a high-performance computing platform at La Trobe University designed for genomic analyses. The assembled contigs were compared against GenBank’s non-redundant nucleotide (BLASTN) and protein (BLASTX) databases [29], using an E-value threshold of 1 × 10^−5^ to minimise false-positive matches. Contigs with significant alignments to bacterial, eukaryotic, or fungal sequences were excluded to retain only viral sequences. Contigs longer than 300 nucleotides were selected for downstream functional analysis in Geneious Prime (version 2023.1.1). The average coverage of the viral contigs was determined using the cleaned raw reads within the same software.

### 2.5. Functional Annotations

The viral genomes, both complete and partial, assembled in this study were annotated using established methodologies [13,15] within Geneious Prime (version 2023.1.1, Biomatters, Ltd., Auckland, New Zealand). Viral taxonomy was determined through comparative analyses utilising GenBank’s BLASTN, BLASTX, and BLASTP tools, with the highest-scoring matches selected based on stringent criteria (E-value < 0.0). Open reading frames (ORFs) within the viral genomes were identified by aligning them with sequences in the NCBI database. Furthermore, the ORFs were analysed against conserved domain databases maintained by the NCBI (Bethesda, MD, USA) [29]. Default software settings were applied unless otherwise specified

### 2.6. Comparative Genomics and Phylogenetic Analyses

Comparative genomic analysis of the newly sequenced viral genomes was performed using Geneious Prime (version 2023.1.1) and Base-By-Base [30]. Sequence similarity between the selected viral sequences and reference viral genomes was assessed through MAFFT alignment (L-INS-I) implemented in Geneious Prime (version 2023.1.1, Biomatters, Ltd., Auckland, New Zealand).

Phylogenetic analyses were conducted using representative viral genomes or conserved gene sequences retrieved from GenBank. Amino acid sequences of selected protein-coding genes were aligned using the MAFFT L-INS-I algorithm within Geneious Prime (version 7.388) [31]. Phylogenetic trees were constructed in Geneious Prime (version 2023.1.1) using RAxML with the Gamma Blosum62 protein model and 1000 bootstrap replicates to ensure robust statistical support. The resulting trees were visualised with FigTree v1.4.4 for interpretation and presentation.

## 3. Results

All the viruses identified in this study were detected in the spleen sample of a dead male platypus (ID: W837-17) found in Apollo Bay, Victoria, Australia. No viral sequence was identified in the samples from the other five dead platypuses.

### 3.1. Evidence of Highly Divergent Papillomaviruses

The four complete genomes of papillomaviruses (PV) identified in the spleen of the platypus were circular double-stranded DNA (dsDNA) genomes, ranging in size from 6100 to 6153 base pairs (bp) (Table 2). The genomes of OaPV1–4 sequenced in this study shared nucleotide sequence identities of 48.90% to 51.22% with a PV genome previously sequenced from the critically endangered axolotl (*Ambystoma mexicanum*) in the United States (GenBank accession no. BK066884.1) (Supplementary Table S1). This was followed by nucleotide identities ranging from 45.68% to 46.61% with PV genomes from cane toads (*Rhinella marina*) in Australia (GenBank accession no. MW582900.1) and 42.60% to 43.55% with PV genomes from canaries (*Serinus canaria*) in Madrid, Spain (GenBank accession no. NC_040548.1). Furthermore, the four PV genomes sequenced in this study exhibited nucleotide identities ranging from 64.91% to 73.32% among themselves, with the highest identity observed between OaPV3 and OaPV4.

Five novel papillomaviruses (OaPV1–5), including a partial genome (OaPV5), were sequenced in this study. All the genomes contained the predicted core methionine-initiated ORFs encoding proteins characteristic of papillomaviruses. These ORFs were annotated as putative genes and numbered sequentially from left to right (Table 2). Comparative analysis of the protein sequences encoded by the predicted ORFs, using BLASTX and BLASTP, identified significant sequence similarities for the L1, E1, and E2 ORFs (Table 2). Interestingly, four of the papillomaviruses (OaPV1–4) contained several hypothetical protein-coding regions unique to this study, as determined by the BLAST database. While the genomes of OaPV1–4 had a ORF of similar sizes as L2 in their expected genomic positions, these proteins did not exhibit significant sequence similarities with known papillomaviruses. Among the predicted protein-coding ORFs of OaPV1–4, the L1 gene displayed the highest amino acid sequence identity, showing notable similarity to the L1 gene of a recently sequenced papillomavirus (papillomavirus ambystoma6078) from the critically endangered axolotl (*Ambystoma mexicanum*) in the United States (Table 2). The predicted E1 and E2 genes also exhibited relatively low amino acid sequence identities, which aligns with the moderate sequence divergence observed at the genomic level. This finding is consistent with the variability typically seen in papillomavirus genomes.

**Figure 1:**
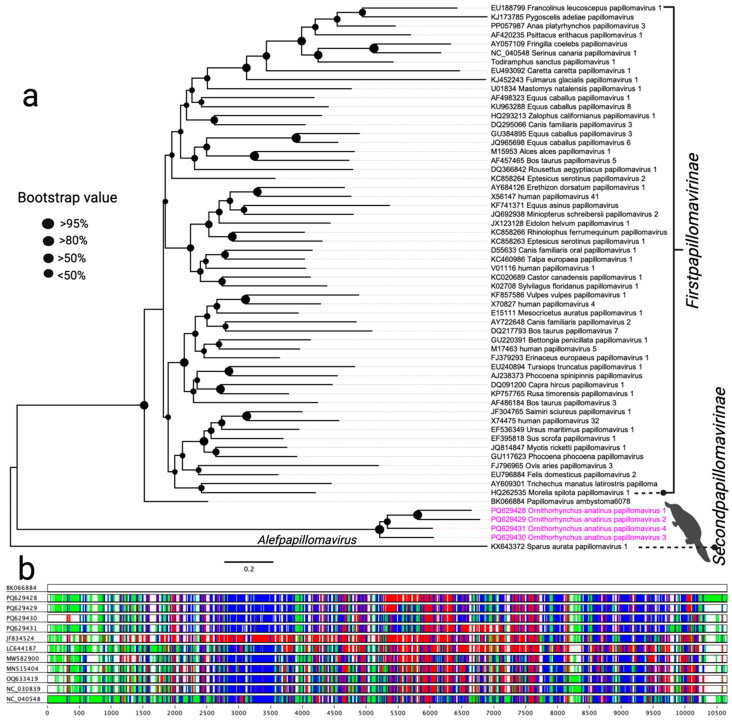
(**a**) Phylogenetic relationships between papillomaviruses detected in this study and other selected PVs. A maximum likelihood (ML) tree was constructed from multiple alignments of the L1 gene using Geneious Prime (version 2023.1.1). The labels at the branch tips refer to GenBank accession numbers, followed by virus names. The positions of the PVs detected in this study are highlighted in pink. (**b**) Visual comparison of L1 gene amino acids from the selected papillomaviruses using Base-By-Base. Differences and indels in the L1 gene between PV sequences in this study and other closely related selected papillomaviruses are shown in different colours (green = insertion, blue = substitution, and red = deletion).

Phylogenetic analysis of the amino acid sequences of the L1 gene supports the classification of the newly identified OaPV1–4 within the subfamily *Secondpapillomavirinae* (Figure 1a). In the resulting maximum likelihood (ML) phylogenetic tree, OaPV1–4 form a distinct subclade positioned between the subclades of two recently described papillomaviruses: one from the critically endangered axolotl (*Ambystoma mexicanum*) and another from the gilt-head bream (*Sparus aurata*) found in the United States and Spain, respectively. Notably, no clear evolutionary connection was observed between the papillomaviruses identified in this study and other known papillomaviruses. This finding suggests that OaPV1–4 may represent an intermediate evolutionary lineage distinct from previously characterised papillomaviruses.

The International Committee on Taxonomy of Viruses (ICTV) classifies papillomaviruses based on nucleotide identity thresholds for the L1 gene, supported by phylogenetic evidence. According to ICTV guidelines, genus, species, and type demarcations are defined by nucleotide identity thresholds of 60%, 70%, and 90%, respectively [32]. Average pairwise identities of the L1 nucleotide sequences for each OaPV type were calculated, as shown in Supplementary Table S2. The L1 nucleotide sequences of all OaPV types exhibited significant divergence from those of previously characterised papillomaviruses (Figure 1b and Supplementary Table S2). Based on this genetic divergence, OaPV1–4 are proposed to belong to a novel genus within the subfamily *Secondpapillomavirinae* that has yet to be formally recognised.

### 3.2. Novel Parvoviruses in Platypus

In this study, five novel parvoviruses were identified, including two with complete genome sequences. These parvoviruses, based on their origin, sequence similarity, and evolutionary relationships, were designated as *Ornithorhynchus anatinus* densovirus 1 and 2 (OaDPV1 and OaDPV2), *Ornithorhynchus anatinus* chaphamaparvovirus 1 (OaChPV1), and *Ornithorhynchus anatinus* parvoviridae species 1 and 2 (OaPV sp1 and OaPV sp2) (Table 3). The genomic structure of the two fully sequenced parvoviruses closely resembles that of other known parvoviruses. Protein sequence comparisons revealed significant alignment (E-values ≤ 10^−5^) for the open reading frames (ORFs), as summarised in Table 3. Notably, the size and positioning of ORF4 in OaDPV1 suggest it is a capsid protein (VP1), yet no significant matches were found in the NCBI database. This aligns with the observation that the parvoviruses detected in this study exhibit high levels of divergence. For instance, the non-structural protein 1 (NS1) sequences of the platypus parvoviruses displayed amino acid identity ranging from 29% to 65% (Table 3). These findings strongly support the classification of these parvoviruses as novel species.

**Figure 2:**
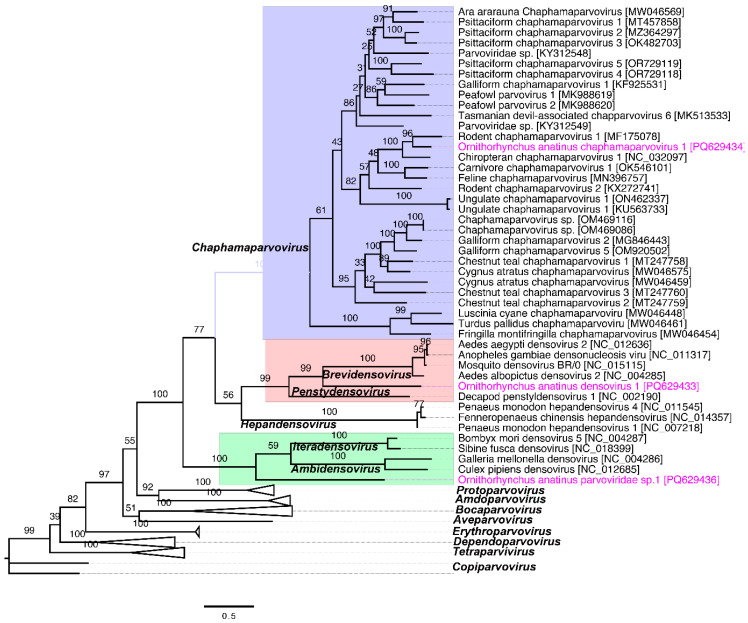
Phylogenetic relationships of parvoviruses detected in this study with other selected parvoviruses. A maximum likelihood (ML) tree was constructed using multiple alignments of the nearly complete NS1 gene amino acid sequences, analysed in Geneious Prime (version 2023.1.1). The branch tip labels denote virus names, followed by their corresponding GenBank accession numbers. Other non-related clades corresponding to this study were collapsed and labelled with the genus name. The parvoviruses identified in this study are highlighted in pink, and relevant clades are marked with distinct colours for better visualisation.

Members of the parvovirus subfamilies are primarily distinguished by their host range, targeting either vertebrates or invertebrates. This classification is strongly supported by phylogenetic analyses based on the amino acid sequence of the viral replication initiator protein (NS1) [33]. Phylogenetic analysis of the NS1 sequences from parvoviruses supports the inclusion of the newly sequenced OaChPV1 within the genus *Chaphamaparvovirus*. This analysis revealed that OaChPV1 is closely related to *Chaphamaparvovirus* species previously identified in wild rats and the common vampire bat (*Desmodus rotundus*) (Figure 2). In contrast, OaDPV1 clustered with members of the genus *Brevidensovirus*, which infect a diverse range of mosquito species (Figure 2). Additionally, *Ornithorhynchus anatinus* parvovirus 1 (OaPV1) was positioned at the root of the parvovirus phylogeny, near the genera *Iteradensovirus* and *Ambidensovirus*. However, it showed no clear evolutionary relationship with other known parvoviruses, suggesting that it may represent a new genus that is yet to be formally established.

### 3.3. Detected Circular DNA Virus

**Figure 3:**
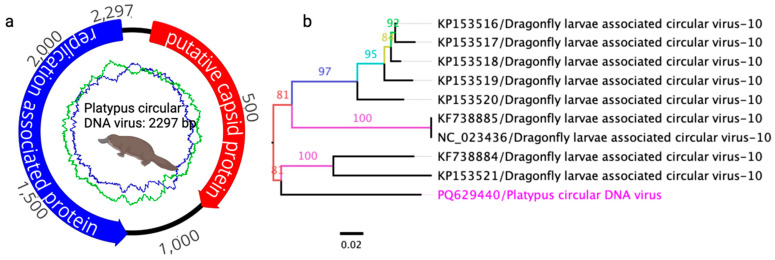
(**a**) Schematic overview of the complete genome of a circular DNA virus sequenced from a platypus. The arrows represent genes and open reading frames, with their orientation indicating the direction of transcription. The blue and green graphs show the GC and AT contents, which are 43.1% and 56.9%, respectively. (**b**) Phylogenetic relationships between the circular DNA virus detected in this study and other selected known circular DNA viruses in GenBank, demonstrating identity with the platypus circular DNA virus. A maximum likelihood (ML) tree was constructed from multiple alignments of complete genome sequences using Geneious Prime (version 2023.1.1). The labels at the branch tips correspond to GenBank accession numbers, followed by virus names. The position of the platypus circular DNA virus detected in this study is highlighted in pink.

A complete genome sequence of a circular DNA virus (2297 nt) was identified in this study (Figure 3a) and deposited in GenBank under accession number PQ629440. The viral genome contains two bidirectional ORFs encoding putative replication-associated and capsid proteins. A BLASTP search of the replication-associated protein sequence revealed the highest identity (97.26%) with a circular DNA virus isolated from an endemic yellow-spotted dragonfly (*Procordulia grayi*) in New Zealand in 2013 (GenBank accession no. ALE29834.1; query coverage: 99%; E-value: 0.0) [34]. Similarly, the capsid protein sequence exhibited 57.78% identity (query coverage: 99%; E-value: 8.0 × 10^−96^) with the same virus (GenBank accession no. ALE29834.1). Phylogenetic analysis of the complete genome sequences from selected circular DNA viruses showed that the virus identified in this study clusters within a distinct subclade alongside the dragonfly larvae-associated circular virus-10, which was also isolated from *Procordulia grayi* in New Zealand (Figure 3b). These findings suggest a shared evolutionary origin for the circular DNA virus sequenced in this study.

### 3.4. Unclassified Tombusviridae

**Figure 4:**
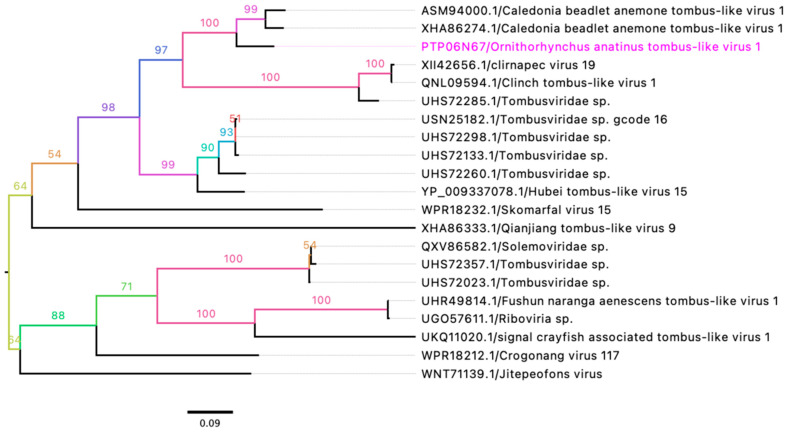
A maximum likelihood (ML) tree was constructed using the amino acid sequences of the putative replicase protein from selected members of the family *Tombusviridae*. The ML tree was generated from multiple alignments of the putative replicase gene sequences using Geneious Prime (version 2023.1.1). The labels at the branch tips indicate GenBank accession numbers followed by virus names. The position of the *Ornithorhynchus anatinus* tombus-like virus 1 (OaTLV1) detected in this study is highlighted in pink.

In this study, two partial genome sequences of novel tombus-like viruses were identified and designated as *Ornithorhynchus anatinus* tombus-like virus 1 (OaTLV1) and *Tombusviridae* sp. These sequences have been deposited in GenBank under accession numbers PQ629438 and PQ629441, respectively (Table 4). The partial genome of OaTLV1 contains three predicted ORFs encoding a putative replicase, capsid protein, and hypothetical protein. Comparative analysis of the encoded protein sequences using BLASTX and BLASTP revealed high sequence similarity between the putative replicase (86.15%) and capsid protein (75.89%) of OaTLV1 and the corresponding proteins of *Caledonia beadlet anemone* tombus-like virus 1 (Table 4). Phylogenetic analysis of the putative replicase sequences from selected tombus-like viruses showed that OaTLV1 clusters within a subclade alongside *Caledonia beadlet anemone* tombus-like virus 1, which was previously detected in swamp crayfish (*Procambarus clarkii*) from China (Figure 4). These findings suggest a shared evolutionary lineage between the two viruses.

### 3.5. Unclassified Nodamuvirales

In this study, a partial genome sequence of a *Nodamuvirus* (3118 nt) was identified and deposited in GenBank under accession number PQ629439. The viral genome contains a single ORF encoding putative hypothetical proteins, which share 99.71% identity with the corresponding proteins of Nelson wasp-associated virus 3 (GenBank accession no. QZZ63401.1), sequenced from the common wasp (*Vespula vulgaris*) in New Zealand in 2006 [35].

### 3.6. Genomoviridae

In this study, a partial genome sequence of a *Genomoviridae* virus (1104 nt) was identified and submitted to GenBank under accession number PQ629442. The viral genome encodes a single ORF for a replication protein catalytic domain-like protein, which shares 97.30% identity with the corresponding protein of *Genomoviridae* sp. (GenBank accession no. XII43235.1). This related sequence was previously obtained from freshwater mussel tissue biopsies in the United States in 2020.

## 4. Discussion

This study represents a significant advancement in understanding viral diversity within monotreme species, uncovering novel and highly divergent viruses detected exclusively in the spleen of a dead male platypus from Apollo Bay, Victoria, Australia. The absence of viral sequences in samples from the other five dead platypuses highlights a potential limitation of the study, as the sampling relied on animals with unknown time of death, which may have impacted viral detection.

Papillomaviruses are a broad group of non-enveloped DNA viruses that infect a wide variety of vertebrate species, including mammals, birds, reptiles, and fish [22,36,37,38,39,40,41]. While many infections are asymptomatic, PVs can also cause benign epithelial growths [22,37,38,39,40]. In this study, we identified four complete genomes of novel papillomaviruses (OaPV1–4) with significant divergence from known sequences (48.90% to 51.22% nucleotide identity compared to axolotl papillomavirus), providing compelling evidence of evolutionary distinctiveness. The observed intra-group nucleotide identity (64.91% to 73.32%) indicates a high level of genomic diversity, suggesting that these PVs could represent a novel genus within the subfamily *Secondpapillomavirinae*. This aligns with studies that document substantial genomic variability within papillomaviruses, contributing to their adaptability and host specificity [42]. Phylogenetic analysis positions OaPV1–4 in a unique subclade between papillomaviruses from axolotls and gilt-head bream, indicating that these viruses may have diverged from a common ancestor but have since evolved distinct lineages. The limited sequence homology of the L1 and L2 genes and the presence of hypothetical protein-coding regions further emphasise their novelty. These findings highlight the potential for monotreme-specific viral evolution and suggest an intermediate evolutionary lineage distinct from known papillomaviruses [43]. Although several papillomaviruses have been reported in healthy wild animals in Australia [44], along with an endogenous papillomavirus from the platypus genome [45], this study was unable to make a connection with the pathological significance of the detected papillomavirus in platypuses, which warrants further investigation.

The *Parvoviridae* family, comprising the *Parvovirinae* and *Densovirinae* subfamilies, includes non-enveloped, single-stranded DNA (ssDNA) viruses that are typically 4–6 kb in length and approximately 25 nm in diameter [46]. In recent years, numerous novel parvoviruses have been identified across diverse hosts, including pigs, rats, and various avian species [12,13,14,47,48,49,50]. However, the pathology of avian parvoviruses and their modes of transmission remain poorly understood. In this study, the discovery of five new parvoviruses, including OaDPV1 and OaDPV2, highlights the complexity of the viral ecosystem within platypuses. The substantial variability observed in NS1 amino acid sequences (29–65% identity) supports their classification as novel species and aligns with previous findings that parvovirus diversity often stems from adaptation to distinct ecological niches [51]. Of particular interest, the relationship between OaChPV1 and chaphamaparvoviruses found in rodents and vampire bats raises the possibility of cross-species transmission or a shared evolutionary origin, warranting further ecological and virological investigation. Additionally, phylogenetic analysis reveals that OaDPV1 clusters within the *Brevidensovirus* genus, which is typically associated with mosquitoes, suggesting an expanded host range for these viruses. Finally, the placement of OaPV1 at the root of the parvovirus phylogeny, near the *Iteradensovirus* and *Ambidensovirus* genera, points to a previously unexplored evolutionary branch. This finding has potential implications for future taxonomic revisions and deepens our understanding of parvovirus evolution [46].

The *Circoviridae* family comprises small, circular, single-stranded DNA (ssDNA) viruses with genome sizes ranging from 1.7 to 2.1 kb. These viruses contain two primary ORFs that encode a replication-associated protein and a capsid protein gene [52,53]. Similarly, circular replication-associated single-stranded (CRESS) DNA viruses found across diverse families such as *Alphasatellitidae*, *Genomoviridae*, and *Circoviridae* share these features [52]. The detection of a complete genome of a circular DNA virus with a high similarity (97.26% identity) to a virus from the yellow-spotted dragonfly (*Procordulia grayi*) is an unexpected finding, highlighting the possibility of shared environmental reservoirs or transmission vectors between aquatic or semi-aquatic species. This supports the notion of viral host adaptability and ecological distribution observed in other studies of circular DNA viruses [34,54]. The clustering of this virus within a subclade of dragonfly-associated circular viruses underscores potential ecological interactions that require further exploration. The partial genome sequence of a *Genomoviridae* virus with significant protein identity (97.30%) to sequences from freshwater mussel biopsies reflects the wide host range and environmental distribution of these viruses. This finding aligns with prior research documenting Genomoviridae in both vertebrate and invertebrate hosts, suggesting environmental reservoirs or shared transmission pathways [55].

The partial genome sequences of OaTLV1 and an unclassified tombus-like virus align closely with tombus-like viruses identified in crayfish, suggesting an aquatic or semi-aquatic viral origin [56]. The high protein sequence similarity (86.15% for replicase) supports the hypothesis of shared evolutionary pathways among viruses found in diverse aquatic hosts. Similarly, the Nodamuvirus detected showed a striking 99.71% protein identity with a virus from common wasps, implying potential cross-order viral transmission or parallel evolutionary adaptations [57,58].

The identification of viruses in the platypus with potential ecological links, such as dragonfly viruses, highlights possible mechanisms of origin and cross-species transmission. Shared aquatic habitats may play a key role, as dragonfly larvae and platypuses coexist in these ecosystems. Viral particles shed by dragonflies into the water could expose platypuses through feeding or contact [59]. Additionally, anthropogenic factors like habitat disturbance and pollution could further increase viral spillover risks by altering ecological dynamics and forcing closer interactions between species [60]. In future studies using more systematic sampling from the platypus, environmental metagenomics and host–virus interaction models are essential to elucidate the mechanisms driving these associations and to assess the risks of emerging viruses in platypuses.

There are several limitations to the genomic and phylogenetic analyses conducted in this study, particularly the limited availability of viral sequence data from monotremes for comparison. This gap in the database poses challenges when analysing highly divergent papillomaviruses and parvoviruses detected in this study. The lack of sequence data from closely related monotreme species hinders our ability to establish accurate evolutionary relationships and assess the broader significance of these findings. Additionally, these results cannot provide insights into prior viral infections or predict the potential impacts of future infections. Moreover, the observed variability in the absence of viruses in the other five platypuses may be attributable to several other factors. One possible explanation is the sample quality, including its storage conditions, handling, and preservation prior to analysis. Poor sample quality can lead to the degradation of nucleic acids, which may impact the accuracy and reproducibility of the results. In addition, methodological constraints cannot be overlooked. Variations in sample processing, such as DNA/RNA extraction efficiencies, differences in reagents or kits, and library preparation methods, could contribute to discrepancies. Future studies should aim to address these potential sources of variability by standardising the sample collection and handling protocols, incorporating metadata, and employing robust analytical methodologies to minimise biases. Moreover, regular diagnostic monitoring is essential to track the evolution and transmission of novel, unique, or significant pathogens. Expanding the viral sequence database through targeted and comprehensive sampling is crucial for more accurate evolutionary analyses, including understanding transmission pathways and viral prevalence within populations.

## 5. Conclusions

The results of this study underscore the remarkable diversity and evolutionary uniqueness of viruses found in the platypus. The high sequence divergence, novel phylogenetic placements, and potential interspecies transmission raise important questions about the ecology, evolution, and host-specific adaptations of these viruses. Continued genomic surveillance and ecological studies are essential to elucidate the role of these viruses in the broader context of wildlife virology and conservation efforts.

## Figures and Tables

**Table 1:** Details of platypus samples used in this study.

Sample ID	Location	Weight (gram)	Sex	History	Gross Path	Sample	Library ID
W837-17	Apollo Bay	1900	male	Barham R	trauma, predation	spleen	PTP06
W653-18	Templestowe	630		on road		liver	PTP03
W1098-19	Strathbogie	1448	male			spleen	PTP05
W475-19	Forest	1500	male	Lake Elizabeth	entanglement, drowned	spleen	PTP02
W354-21	Bright	979	female			spleen	PTP04
W1057-22	Gunbower			on road		spleen	PTP01

**Table 2:** Detected PV genome annotations and comparative analysis of ORFs.

Gene Synteny	Genome Coordinates	nt Size	AA Size	Best Blast Hits (GenBank Accession Number)	Product	Similarity (%)	Note
***Ornithorhynchus anatinus* papillomavirus 1 (OaPV1, GenBank accession no. PQ629428), length-6153 bp, complete genome**							
ORF1	170–808	639	212	no significant Blast hit			hypothetical gene
ORF2	258–638	381	126	no significant Blast hit			hypothetical gene
ORF3	811–2313	1503	500	Papillomavirus ambystoma6078 (DBA51064.1)	E1	45.12	
ORF4	2261–3115	855	284	Sparus aurata papillomavirus 1 (YP_009272701.1)	E2	26.05	
ORF5	3144–3428	285	94	no significant Blast hit			hypothetical gene
ORF6	3422–4045	624	207	no significant Blast hit			hypothetical gene
ORF7	4045–5502	1458	485	Papillomavirus ambystoma6078 (DBA51069.1)	L1	37.52	
***Ornithorhynchus anatinus* papillomavirus 2 (OaPV2, GenBank accession no. PQ629429), length-6151 bp, complete genome**							
ORF1	168–779	612	203	no significant Blast hit			hypothetical gene
ORF2	256–630	375	124	no significant Blast hit			hypothetical gene
ORF3	781–2280	1500	499	Papillomavirus ambystoma6078 (DBA51064.1)	E1	40	
ORF4	2228–3100	873	290	Papillomavirus sparus5907 (DBA50395.1)	E2	25.09	
ORF5	3111–3713	603	200	no significant Blast hit			hypothetical gene
ORF6	3332–4360	1029	342	no significant Blast hit			hypothetical gene
ORF7	4360–5841	1482	493	Papillomavirus ambystoma6078 (DBA51069.1)	L1	39.17	
***Ornithorhynchus anatinus* papillomavirus 3 (OaPV3, GenBank accession no. PQ629430), length-6104 bp, complete genome**							
ORF1	61–351	291	96	no significant Blast hit			hypothetical gene
ORF2	509–2023	1515	504	Papillomavirus ambystoma6078 (DBA51064.1)	E1	40.08	
ORF3	1956–2801	846	281	no significant Blast hit			hypothetical gene
ORF4	2801–4243	1443	480	no significant Blast hit			hypothetical gene
ORF5	4243–5694	1452	483	Papillomavirus ambystoma6078 (DBA51069.1)	L1	41.13	
***Ornithorhynchus anatinus* papillomavirus 4 (OaPV4, GenBank accession no. PQ629431), length-6100 bp, complete genome**							
ORF1	148–780	633	210	no significant Blast hit			hypothetical gene
ORF2	236–613	378	125	no significant Blast hit			hypothetical gene
ORF3	782–2311	1530	509	Papillomavirus ambystoma6078 (DBA51064.1)	E1	46.45	
ORF4	2244–3068	825	274	no significant Blast hit			hypothetical gene
ORF5	3068–4027	960	319	no significant Blast hit			hypothetical gene
ORF6	4027–5502	1476	491	Papillomavirus ambystoma6078 (DBA51069.1)	L1	38.54	
***Ornithorhynchus anatinus* papillomavirus 5 (OaPV5, GenBank accession no. PQ629432), length-1135 bp, partial cds**							
ORF3	161–1072	912	303	Papillomavirus ambystoma6078 (DBA51064.1)	E1	49.29	

**Table 3:** Detected parvoviruses genome annotations and comparative analysis of ORFs.

Gene Synteny	Genome Coordinates	nt Size	AA Size	Best Blast Hits (GenBank Accession Number)	Product	Similarity (%)	Note
***Ornithorhynchus anatinus* densovirus 1 (OaDPV1, GenBank accession no. PQ629433), length-4639 bp, complete genome**							
ORF1	220–423	204	67	no significant Blast hit			hypothetical gene
ORF2	360–2699	2340	779	Ambidensovirus sp. (AVM80379.1)	NS1	32.32	
ORF3	449–997	549	182	Aedes vexans densovirus (UTQ11533.1)	NS2	33.33	
ORF4	2783–4090	1308	435	no significant Blast hit			hypothetical gene
***Ornithorhynchus anatinus* chaphamaparvovirus 1 (OaChPV1, GenBank accession no. PQ629434), length-4000 bp, complete genome**							
ORF1	34–288	255	84	Psittacine parvovirus 1 (YP_010805269.1)	hypothetical gene	43.75	
ORF2	285–695	411	136	Mouse kidney parvovirus (QLM06160.1)	NS3	71.56	
ORF3	596–2584	1989	662	Bat chaphamaparvovirus (QOR29549.1)	NS1	65.21	
ORF4	1869–2522	654	217	Mouse kidney parvovirus (AXX39021.1)	NS2	69.12	
ORF5	2577–3956	1380	459	Bat chaphamaparvovirus (QOR29550.1)	VP1	72.08	
***Ornithorhynchus anatinus* densovirus 2 (OaDPV2, GenBank accession no. PQ629435), length-1612 bp, partial cds**							
ORF1	33–638	606	201	Parvoviridae sp. (WAQ80633.1)	NS	41.41	
ORF2	1222–1566	345	114	Turdus hortulorum ambidensovirus (QTE04092.1)	NS	29.27	
***Ornithorhynchus anatinus* parvoviridae sp.1 (OaPV sp1, GenBank accession no. PQ629436), length-2146 bp, partial cds**							
ORF1	119–1906	1788	595	Periparus ater parvoviridae sp. (QTE03714.1)	NS1	40.00	
***Ornithorhynchus anatinus* parvoviridae sp.2 (OaPV sp2, GenBank accession no. PQ629437), length-1077 bp, partial cds**							
ORF1	50–844	795	264	Periparus ater parvoviridae sp. (QTE03714.1)	NS1	49.19	

**Table 4:** Detected *Tombusviridae* annotations and comparative analysis of the ORFs.

Gene Synteny	Genome Coordinates	nt Size	AA Size	Best Blast Hits (GenBank Accession Number)	Product	Similarity (%)
***Ornithorhynchus anatinus* tombus-like virus 1 (OaTLV1, GenBank accession no. PQ629438), length-3944 bp, partial genome**						
ORF1	40–1248	1209	402	putative hypothetical protein (ASM93999.1)	hypothetical protein	58.35
ORF2	1660–2742	1083	360	putative replicase (ASM94000.1)	replicase	86.15
ORF3	2748–3821	1074	357	putative capsid (ASM94001.1)	capsid	75.89
***Ornithorhynchus anatinus* Tombusviridae sp. (GenBank accession no. PQ629441), length-1201 bp, partial genome**						
ORF1	519–1133	615	204	putative coat protein (UHS72286.1)	capsid	32.68

## Data Availability

The nucleotide sequences and associated data from this study are available in the DDBJ/EMBL/GenBank databases under accession numbers PQ629428–PQ629442.

## Supplementary Materials

The following supporting information can be downloaded at https://www.mdpi.com/article/10.3390/v17020176/s1: Table S1: Nucleotide identity of the selected papillomaviruses at the genomic level; Table S2: Nucleotide identity of the selected papillomaviruses’ NS1 gene.

## References

[1] Warren W., Hillier L., Marshall Graves J., Birney E., Ponting C., Grützner F., Belov K., Miller W., Clarke L., Chinwalla A. (2008). Genome analysis of the platypus reveals unique signatures of evolution. Nature.

[2] Bino G., Kingsford R.T., Archer M., Connolly J.H., Day J., Dias K., Goldney D., Gongora J., Grant T., Griffiths J. (2019). The platypus: Evolutionary history, biology, and an uncertain future. J. Mammal..

[3] Zhou Y., Shearwin-Whyatt L., Li J., Song Z., Hayakawa T., Stevens D., Fenelon J.C., Peel E., Cheng Y., Pajpach F. (2021). Platypus and echidna genomes reveal mammalian biology and evolution. Nature.

[4] Wille M., Shi M., Klaassen M., Hurt A.C., Holmes E.C. (2019). Virome heterogeneity and connectivity in waterfowl and shorebird communities. ISME J..

[5] Sutherland M., Sarker S., Vaz P.K., Legione A.R., Devlin J.M., Macwhirter P.L., Whiteley P.L., Raidal S.R. (2019). Disease surveillance in wild Victorian cacatuids reveals co-infection with multiple agents and detection of novel avian viruses. Vet. Microbiol..

[6] Wille M., Eden J.S., Shi M., Klaassen M., Hurt A.C., Holmes E.C. (2018). Virus-virus interactions and host ecology are associated with RNA virome structure in wild birds. Mol. Ecol..

[7] Vibin J., Chamings A., Klaassen M., Bhatta T.R., Alexandersen S. (2020). Metagenomic characterisation of avian parvoviruses and picornaviruses from Australian wild ducks. Sci. Rep..

[8] Vibin J., Chamings A., Collier F., Klaassen M., Nelson T.M., Alexandersen S. (2018). Metagenomics detection and characterisation of viruses in faecal samples from Australian wild birds. Sci. Rep..

[9] Shi M., Lin X.D., Tian J.H., Chen L.J., Chen X., Li C.X., Qin X.C., Li J., Cao J.P., Eden J.S. (2016). Redefining the invertebrate RNA virosphere. Nature.

[10] White R.T., Taylor W., Klukowski N., Vaughan-Higgins R., Williams E., Petrovski S., Rose J.J.A., Sarker S. (2023). A discovery down under: Decoding the draft genome sequence of Pantoea stewartii from Australia’s critically endangered western ground parrot/kyloring (*Pezoporus flaviventris*). Microb. Genom..

[11] White R.T., Jelocnik M., Klukowski N., Haque M.H., Sarker S. (2023). The first genomic insight into Chlamydia psittaci sequence type (ST)24 from a healthy captive psittacine host in Australia demonstrates evolutionary proximity with strains from psittacine, human, and equine hosts. Vet. Microbiol..

[12] Klukowski N., Eden P., Uddin Muhammad J., Sarker S. (2023). Virome of Australia’s most endangered parrot in captivity evidenced of harboring hitherto unknown viruses. Microbiol. Spectr..

[13] Sarker S., Talukder S., Anwar A., Van T.T., Petrovski S. (2022). Unravelling Bile Viromes of free-range laying chickens clinically diagnosed with spotty liver disease: Emergence of many novel chaphamaparvoviruses into multiple lineages. Viruses.

[14] Sarker S. (2021). Molecular and phylogenetic characterisation of a highly divergent novel parvovirus (psittaciform chaphamaparvovirus 2) in australian neophema parrots. Pathogens.

[15] Sarker S. (2021). Metagenomic detection and characterisation of multiple viruses in apparently healthy Australian Neophema birds. Sci. Rep..

[16] Geoghegan J.L., Duchêne S., Holmes E.C. (2017). Comparative analysis estimates the relative frequencies of co-divergence and cross-species transmission within viral families. PLoS Pathog..

[17] Ng T.F.F., Manire C., Borrowman K., Langer T., Ehrhart L., Breitbart M. (2009). Discovery of a novel single-stranded DNA virus from a sea turtle fibropapilloma by using viral metagenomics. J. Virol..

[18] Tisza M.J., Pastrana D.V., Welch N.L., Stewart B., Peretti A., Starrett G.J., Pang Y.S., Krishnamurthy S.R., Pesavento P.A., McDermott D.H. (2020). Discovery of several thousand highly diverse circular DNA viruses. Elife.

[19] Cui X., Fan K., Liang X., Gong W., Chen W., He B., Chen X., Wang H., Wang X., Zhang P. (2023). Virus diversity, wildlife-domestic animal circulation and potential zoonotic viruses of small mammals, pangolins and zoo animals. Nat. Commun..

[20] Frias-De-Diego A., Jara M., Escobar L.E. (2019). Papillomavirus in Wildlife. Front. Ecol. Evol..

[21] Rector A., Van Ranst M. (2013). Animal papillomaviruses. Virology.

[22] Mifsud J.C.O., Hall J., Van Brussel K., Rose K., Parry R.H., Holmes E.C., Harvey E. (2024). A novel papillomavirus in a New Zealand fur seal (*Arctocephalus forsteri*) with oral lesions. Npj Viruses.

[23] Daszak P., Cunningham A.A., Hyatt A.D. (2001). Anthropogenic environmental change and the emergence of infectious diseases in wildlife. Acta Trop..

[24] Shi M., Zhang Y.Z., Holmes E.C. (2018). Meta-transcriptomics and the evolutionary biology of RNA viruses. Virus Res..

[25] Athukorala A., Phalen D.N., Das A., Helbig K.J., Forwood J.K., Sarker S. (2021). Genomic characterisation of a highly divergent siadenovirus (psittacine siadenovirus f) from the critically endangered orange-bellied parrot (*Neophema chrysogaster*). Viruses.

[26] Sarker S., Das S., Lavers J.L., Hutton I., Helbig K., Imbery J., Upton C., Raidal S.R. (2017). Genomic characterization of two novel pathogenic avipoxviruses isolated from pacific shearwaters (*Ardenna* spp.). BMC Genom..

[27] Sarker S., Isberg R.S., Moran L.J., Araujo D.R., Elliott N., Melville L., Beddoe T., Helbig J.K. (2019). Crocodilepox Virus evolutionary genomics supports observed poxvirus infection dynamics on saltwater crocodile (*Crocodylus porosus*). Viruses.

[28] Bankevich A., Nurk S., Antipov D., Gurevich A.A., Dvorkin M., Kulikov A.S., Lesin V.M., Nikolenko S.I., Pham S., Prjibelski A.D. (2012). SPAdes: A new genome assembly algorithm and its applications to single-cell sequencing. J. Comput. Biol. J. Comput. Mol. Cell Biol..

[29] Benson D.A., Cavanaugh M., Clark K., Karsch-Mizrachi I., Lipman D.J., Ostell J., Sayers E.W. (2013). GenBank. Nucleic Acids Res..

[30] Hillary W., Lin S.-H., Upton C. (2011). Base-By-Base version 2: Single nucleotide-level analysis of whole viral genome alignments. Microb. Inform. Exp..

[31] Katoh K., Standley D.M. (2013). MAFFT Multiple Sequence Alignment Software Version 7: Improvements in Performance and Usability. Mol. Biol. Evol..

[32] Van Doorslaer K., Chen Z., Bernard H.U., Chan P.K.S., DeSalle R., Dillner J., Forslund O., Haga T., McBride A.A., Villa L.L. (2018). ICTV Virus Taxonomy Profile: Papillomaviridae. J. Gen. Virol..

[33] Cotmore S.F., Agbandje-McKenna M., Canuti M., Chiorini J.A., Eis-Hubinger A.-M., Hughes J., Mietzsch M., Modha S., Ogliastro M., Pénzes J.J. (2019). ICTV Virus Taxonomy Profile: Parvoviridae. J. Gen. Virol..

[34] Dayaram A., Galatowitsch M.L., Argüello-Astorga G.R., van Bysterveldt K., Kraberger S., Stainton D., Harding J.S., Roumagnac P., Martin D.P., Lefeuvre P. (2016). Diverse circular replication-associated protein encoding viruses circulating in invertebrates within a lake ecosystem. Infect. Genet. Evol..

[35] Remnant E.J., Baty J.W., Bulgarella M., Dobelmann J., Quinn O., Gruber M.A.M., Lester P.J. (2021). A diverse viral community from predatory wasps in their native and invaded range, with a new virus infectious to honey bees. Viruses.

[36] Varsani A., Kraberger S., Jennings S., Porzig E.L., Julian L., Massaro M., Pollard A., Ballard G., Ainley D.G. (2014). A novel papillomavirus in Adélie penguin (*Pygoscelis adeliae*) faeces sampled at the Cape Crozier colony, Antarctica. J. Gen. Virol..

[37] Nicholls P.K., Stanley M.A. (2000). The immunology of animal papillomaviruses. Vet. Immunol. Immunopathol..

[38] Campo M.S. (2003). Papillomavirus and disease in humans and animals. Vet. Comp. Oncol..

[39] Cubie H.A. (2013). Diseases associated with human papillomavirus infection. Virology.

[40] Canuti M., Munro H.J., Robertson G.J., Kroyer A.N.K., Roul S., Ojkic D., Whitney H.G., Lang A.S. (2019). New insight into avian papillomavirus ecology and evolution from characterization of novel wild bird papillomaviruses. Front. Microbiol..

[41] Olivo D., Kraberger S., Varsani A. (2024). New duck papillomavirus type identified in a mallard in Missouri, USA. Arch. Virol..

[42] Van Doorslaer K. (2013). Evolution of the Papillomaviridae. Virology.

[43] de Villiers E.M., Fauquet C., Broker T.R., Bernard H.U., zur Hausen H. (2004). Classification of papillomaviruses. Virology.

[44] Antonsson A., McMillan N.A.J. (2006). Papillomavirus in healthy skin of Australian animals. J. Gen. Virol..

[45] Cui J., Holmes E.C. (2012). Evidence for an endogenous papillomavirus-like element in the platypus genome. J. Gen. Virol..

[46] Pénzes J.J., Söderlund-Venermo M., Canuti M., Eis-Hübinger A.M., Hughes J., Cotmore S.F., Harrach B. (2020). Reorganizing the family Parvoviridae: A revised taxonomy independent of the canonical approach based on host association. Arch. Virol..

[47] Shan T., Yang S., Wang H., Wang H., Zhang J., Gong G., Xiao Y., Yang J., Wang X., Lu J. (2022). Virome in the cloaca of wild and breeding birds revealed a diversity of significant viruses. Microbiome.

[48] Zhang Y., Talukder S., Bhuiyan M.S.A., He L., Sarker S. (2024). Opportunistic sampling of yellow canary (*Crithagra flaviventris*) has revealed a high genetic diversity of detected parvoviral sequences. Virology.

[49] Sarker S., Athukorala A., Phalen D.N. (2022). Characterization of a near-complete genome sequence of a chaphamaparvovirus from an australian boobook owl (*Ninox boobook*). Microbiol. Resour. Announc..

[50] Sarker S. (2022). Characterization of a novel complete-genome sequence of a galliform chaphamaparvovirus from a free-range laying chicken clinically diagnosed with spotty liver disease. Microbiol. Resour. Announc..

[51] Pérez-Losada M., Arenas M., Galán J.C., Palero F., González-Candelas F. (2015). Recombination in viruses: Mechanisms, methods of study, and evolutionary consequences. Infect. Genet. Evol..

[52] Breitbart M., Delwart E., Rosario K., Segalés J., Varsani A., Ictv Report C. (2017). ICTV Virus Taxonomy Profile: Circoviridae. J. Gen. Virol..

[53] Sarker S., Terron M.C., Khandokar Y., Aragao D., Hardy J.M., Radjainia M., Jimenez-Zaragoza M., de Pablo P.J., Coulibaly F., Luque D. (2016). Structural insights into the assembly and regulation of distinct viral capsid complexes. Nat. Commun..

[54] Rosario K., Mettel K.A., Benner B.E., Johnson R., Scott C., Yusseff-Vanegas S.Z., Baker C.C.M., Cassill D.L., Storer C., Varsani A. (2018). Virus discovery in all three major lineages of terrestrial arthropods highlights the diversity of single-stranded DNA viruses associated with invertebrates. PeerJ.

[55] Varsani A., Krupovic M. (2021). Family Genomoviridae: 2021 taxonomy update. Arch. Virol..

[56] Zhang Q.-Y., Ke F., Gui L., Zhao Z. (2022). Recent insights into aquatic viruses: Emerging and reemerging pathogens, molecular features, biological effects, and novel investigative approaches. Water Biol. Secur..

[57] Vasilakis N., Tesh R.B. (2015). Insect-specific viruses and their potential impact on arbovirus transmission. Curr. Opin. Virol..

[58] Mayer S.V., Tesh R.B., Vasilakis N. (2017). The emergence of arthropod-borne viral diseases: A global prospective on dengue, chikungunya and zika fevers. Acta Trop..

[59] Longdon B., Brockhurst M.A., Russell C.A., Welch J.J., Jiggins F.M. (2014). The evolution and genetics of virus host shifts. PLoS Pathog..

[60] Kock R.A., Woodford M.H., Rossiter P.B. (2010). Disease risks associated with the translocation of wildlife. Rev. Sci. Tech. Int. Off. Epizoot..

